# Pharmacological Evaluation of Polygoni Multiflori Radix Praeparata Extract: Inhibition of PANoptosis in Alleviating Premature Ovarian Insufficiency

**DOI:** 10.3390/cimb47070569

**Published:** 2025-07-19

**Authors:** Can Zhu, Jinhong Li, Yaofeng Li, Daiyong Chen, Chang Lin

**Affiliations:** 1 College of Basic Medicine, Guizhou University of Traditional Chinese Medicine, Guiyang 550025, China; 2 College of Basic Chinese Medicine, Guizhou University of Traditional Chinese Medicine, Guiyang 550025, China; 3 College of Pharmacy, Guizhou University of Traditional Chinese Medicine, Guiyang 550025, China

**Keywords:** polygoni multiflori radix praeparata, premature ovarian insufficiency, PANoptosis, PANoptosome

## Abstract

Polygoni Multiflori Radix Praeparata (PMRP), a processed root of *Polygonum multiflorum* Thunb. (known as Zhiheshouwu in Chinese medicine), exhibits anti-aging properties and is used to improve ovarian aging. However, its therapeutic mechanism against premature ovarian insufficiency (POI) remains unclear. This study investigates whether PMRP alleviates POI by inhibiting PANoptosis—a cell death pathway characterized by the concurrent occurrence and interplay of pyroptosis, apoptosis, and necroptosis. POI was induced in rats using tripterygium glycosides. We evaluated the estrous cycle, serum hormone levels (follicle-stimulating hormone [FSH], estrogen [E_2_], anti-Müllerian hormone [AMH]), follicular development, and the ultrastructure of granulosa cells. PANoptosome assembly (apoptosis-associated speck-like protein containing a CARD [ASC]/caspase-8/receptor-interacting protein kinase 3 [RIPK3] co-localization) and key effectors of PANoptosis (caspase 3, cleaved caspase 3, gasdermin D [GSDMD], cleaved GSDMD, GSDME, RIPK1, mixed-lineage kinase domain-like protein [MLKL], and p-MLKL) were analyzed. PMRP restored the estrous cycle, lowered FSH levels, and increased E_2_ and AMH levels in POI rats. It reduced follicular atresia, preserved primordial follicles, and suppressed PANoptosis-like death in granulosa cells. Mechanistically, PMRP disrupted PANoptosome assembly and downregulated key effectors of PANoptosis. PMRP alleviates POI by inhibiting PANoptosis in granulosa cells, overcoming the previous limitations of targeting single death pathways and providing novel insights into the pathogenesis and treatment strategies for POI.

## 1. Introduction

Premature ovarian insufficiency (POI), defined as the cessation of ovarian function before the age of 40, is characterized by oligomenorrhea or amenorrhea, elevated follicle-stimulating hormone (FSH) levels, and decreased estrogen (E_2_) levels, with the advanced stage termed premature ovarian failure (POF) [1,2]. This condition not only results in infertility and reduced sexual function, but also leads to perimenopausal symptoms and increases the risks for osteoporosis, cognitive impairment, and cardiovascular diseases, significantly affecting patients’ quality of life and posing societal burdens. The pathogenesis of POI involves immune, genetic, iatrogenic, environmental, and psychosocial factors, though over 50% of cases remain idiopathic. Exploring complex cell death pathways like PANoptosis may shed light on these idiopathic cases. Research indicates that ovarian dysfunction is associated with a low-grade inflammatory microenvironment [3]. For instance, proteins related to ovarian pyroptosis and apoptosis are significantly upregulated in radiation-induced POI mice [4], while granulosa cells from POI models under endoplasmic reticulum stress exhibit features of necroptosis and apoptosis [5]. This suggests that multiple cell death pathways may collectively contribute to ovarian dysfunction. PANoptosis, a recently identified form of inflammatory programmed cell death described in 2019, is characterized by the concurrent occurrence and interplay of pyroptosis, apoptosis, and necroptosis [6,7]. This process is regulated by a polymeric protein complex known as the PANoptosome, which includes key factors associated with each cell death pathway, and activates downstream effectors such as caspase 3/7, gasdermin D/E (GSDMD/E), and mixed-lineage kinase domain-like protein (MLKL), ultimately leading to inflammatory cell death [8]. Therefore, PANoptosis may play a crucial role in the pathogenesis of POI. A recent study has indicated that short-chain chlorinated paraffins can induce ovarian damage in mice through the absent in melanoma 2 (AIM2) and NOD-like receptors family pyrin domain containing 12 (NLRP12)-PANoptosome pathways [9]; however, the current understanding of the relationship between PANoptosis and the mechanisms underlying POI is still in its infancy.

Current management of POI primarily depends on hormone replacement therapy and assisted reproductive technology, which fail to address the underlying causes and may increase the risks of venous thromboembolism, breast cancer, and endometrial cancer with long-term use [10,11]. These limitations underscore the need for safer and more effective therapeutic alternatives. Traditional Chinese medicine offers unique advantages in the treatment of POI through its multi-target approach, fewer side effects, and lower costs. Polygoni Multiflori Radix Praeparata (PMRP), commonly known as Zhiheshouwu, and renowned for its anti-aging properties in Chinese medicine [12], is a processed product derived from the dried root tuber of *Polygonum multiflorum* Thunb. (the plant name has been checked using http://www.theplantlist.org, accessed on 19 April 2025).

According to the *Bencao Mengquan*, PMRP is praised for its ability to “nourish tendons and bones, enhance complexion, improve blood and qi, and promote fertility with long-term use.” Modern pharmacological studies have confirmed its anti-aging, anti-inflammatory, immunomodulatory, and antioxidant properties [13,14,15], making it a promising candidate for preventing and treating aging-related diseases. Notably, PMRP has demonstrated the capacity to normalize sex hormone levels and ameliorate ovarian dysfunction induced by tripterygium glycosides in rats [16]. Its phytoestrogen-like effects are evidenced by enhanced uterine growth and increased estrogen receptor expression in juvenile mice [17]. The major active ingredient, 2,3,5,4′-tetrahydroxystilbene-2-O-β-D-glucoside (TSG), has been shown to protect the ovaries of naturally aged mice from stress-induced damage and to enhance the expression of genes involved in steroid hormone production [18]. However, whether PMRP exerts its therapeutic effects through modulation of PANoptosis remains unknown. This study aims to establish POI models to investigate the mechanisms by which granulosa cell PANoptosis leads to ovarian dysfunction, and to elucidate how PMRP may regulate PANoptosis to mitigate follicular atresia, thereby protecting the ovarian function. By overcoming the previous limitations of targeting single death pathways, this research may provide novel insights for further exploration of the pathogenesis and therapeutic strategies for POI.

## 2. Materials and Methods

### 2.1. Materials

Tripterygium glycosides (TG, Cat# 20240701) were purchased from Hunan Qianjin Xieli Pharmaceutical Co., Ltd. (Zhuzhou, China). Estradiol valerate (Cat# 821A) was acquired from Bayer HealthCare GmbH (Leverkusen, Germany). Enzyme-linked immunosorbent assay (ELISA) kits for rat FSH (Cat# E-OSEL-R0001), E_2_ (Cat# E-EL-R0391), and anti-Müllerian hormone (AMH, Cat# E-EL-R3022) were sourced from Elabscience Biotechnology Co., Ltd. (Wuhan, China). Primary antibodies included rabbit anti-ASC (Cat# DF6304), anti-caspase 8 (Cat# AF6442), anti-GAPDH (Cat# AF7021), and anti-phospho-MLKL (Cat# AF7420) from Affinity Biosciences (Changzhou, China); rabbit anti-caspase 3 (Cat# A2156), anti-GSDMD (Cat# A24476), anti-GSDME (Cat# A7432), anti-RIPK3 (Cat# A5431), and anti-MLKL (Cat# A17312) from ABclonal Biotechnology Co., Ltd. (Wuhan, China); and rabbit anti-RIPK1 (Cat# 29932-1-AP) and mouse anti-cleaved caspase 3 (Cat# 68773-1-Ig) from Proteintech Group, Inc. (Wuhan, China).

### 2.2. Preparation of PMRP

PMRP (batch 240301) was provided by Beijing Bencao Fangyuan Pharmaceutical Technology Co., Ltd. (Beijing, China). The decoction was prepared by soaking PMRP in distilled water at a ratio of ten times its weight for 30 min, followed by boiling for 60 min. The filtrate was collected, and the residue was subjected to additional boiling with eight times its weight in water for 40 min to obtain a second filtrate. The two filtrates were then combined and concentrated to yield crude drug solutions at 0.1 g/mL and 0.4 g/mL for further use.

### 2.3. Component Analysis of PMRP

PMRP (0.1 g) was dissolved in 200 µL of water, followed by methanol extraction (1 mL) with 10 min of grinding and 10 min of vortexing. After centrifugation (13,000 rpm, 10 min), the supernatant was analyzed via liquid chromatography-tandem mass spectrometry (LC-MS/MS). The high-resolution liquid mass spectrometry data were initially processed using Compound Discoverer 3.3 (Thermo Fisher Scientific, Massachusetts, Waltham, USA) and subsequently compared to the mzCloud database for identification. For standard comparison and content determination, TSG (batch J1708Z45941, 98% purity), Emodin (batch M29IB216001, 98% purity), and Emodin-3-methyl ether (batch JT28617A, 98% purity) were obtained from Yuanye Biotechnology Co., Ltd. (Shanghai, China). Chromatogram acquisition and integration were accomplished using Xcalibur 4.0 software.

### 2.4. POI Rat Model and Treatment

Female Sprague-Dawley rats (200–220 g) were purchased from Henan Sike Beisi Biotechnology Co., Ltd. (Anyang, China) and maintained under controlled conditions (12 h light/dark cycle, 22 ± 1 °C) with free access to food and water. All experimental protocols adhered to internationally accepted principles for laboratory animal care, such as those outlined in the European Community guidelines (EEC Directive of 1986; 86/609/EEC), and were approved by the Animal Ethics Committee of Guizhou University of Traditional Chinese Medicine (No. 20241013001).

Rats with normal ovarian function were selected through vaginal cytology screening, and were randomly assigned to five groups (*n* = 6, a total of 30). The control group received distilled water via gavage, while the model group was administered with tripterygium glycosides (TG, 75 mg/kg/d) to induce POI [19,20]. The estradiol valerate (EV) group, serving as the positive control, received both TG (75 mg/kg/d) and EV (0.18 mg/kg/d). Based on previous studies [16], the PMRP low-dose (PMRP-L) group was treated with TG (75 mg/kg/d) and PMRP (1 g/kg/d, equivalent to the clinical human equivalent dose), while the PMRP high-dose (PMRP-H) group received TG (75 mg/kg/d) and PMRP (4 g/kg/d, 4 times to the clinical human equivalent dose). All treatments were administered for 28 consecutive days.

### 2.5. Estrous Cycle Evaluation and Ovarian Index Measurement

Daily vaginal cytological examinations were performed throughout the intervention period to monitor estrous cycle [21]. The procedure involved gently introducing a saline-moistened cotton swab into the vaginal canal, followed by rotation and subsequent withdrawal. The collected cellular material was transferred to glass slides for microscopic examination, with cycle phases were determined based on characteristic cellular morphology. On the day after the last administration, blood and ovaries were collected, and the ovarian index was calculated as the ratio of ovarian weight to body weight (ovarian index = ovarian weight/body weight × 100%).

### 2.6. ELISA

Blood samples were allowed to stand at room temperature for 1 h, followed by centrifugation at 2000 rpm for 10 min to obtain the supernatant. Serum concentrations of FSH, E_2_, and AMH were quantified using commercial ELISA kits, following the manufacturer’s protocols. Optical density measurements were performed at the specified wavelength for each assay, with hormone concentrations determined through standard curves.

### 2.7. Hematoxylin–Eosin (HE) Staining

Ovarian tissues were fixed in 4% paraformaldehyde, followed by standard tissue processing involving graded ethanol dehydration, xylene clearing, paraffin embedding, and slicing procedures. The tissue sections were then dewaxed and stained with hematoxylin and eosin. After dehydration, the sections were sealed for observation. The morphological structure of the ovarian tissues was examined under a light microscope (Nikon Eclipse Ci-L, Tokyo, Japan), and images were captured for analysis. Follicles at different developmental stages were classified and counted, as follows: primordial follicles (oocytes surrounded by a single layer of flattened granulosa cells); primary follicles (oocytes enclosed by one or more layers of cuboidal/columnar granulosa cells, with a visible zona pellucida); secondary follicles (primary follicles with antral cavities); mature follicles (large, thin-walled follicles protruding toward the ovarian surface); atretic follicles (exhibiting oocyte/granulosa cell degeneration, zona pellucida collapse, and interstitial gland formation); and corpus luteum (a vascularized endocrine structure formed post-ovulation from the collapsed follicular cavity) [22].

### 2.8. Transmission Electron Microscopy (TEM)

Ovarian cortical specimens (1 mm^3^) were immediately pre-fixed in a buffer solution containing 2% paraformaldehyde and 2.5% glutaraldehyde, followed by post-fixation with a solution of 1% osmium tetroxide. The specimens underwent dehydration, were stained with gelatin, and then were polymerized and embedded before being sliced into 0.8 μm sections. Toluidine blue staining was performed to identify the area of the growing follicle wall, which was subsequently sectioned into ultrathin slices (90 nm). Uranium acetate–lead citrate electron staining was applied, and the specimens were dried. Images were captured using a TEM microscope (Hitachi HT7800, Tokyo, Japan).

### 2.9. Immunofluorescence

Paraffin sections of ovarian tissue were dewaxed and rehydrated. Antigen retrieval was performed, followed by blocking of endogenous peroxidase and serum. Primary antibodies (ASC at 1:200, caspase 8 at 1:200, RIPK3 at 1:100) were added and incubated overnight at 4 °C. Following this, secondary antibodies were applied and incubated at room temperature, and DAPI was used to counterstain the cell nuclei before the sections were sealed. For each group, ovarian sections from three randomly selected rats were analyzed. Fluorescence images were captured using a CX40 microscope (SOPTOP, Yuyao, China) with at least three randomly selected fields per section, focusing on follicular structures. Co-localization of ASC, caspase-8, and RIPK3 were observed. Quantitative analysis was performed using Image-J 1.52i software (Bio-Rad, Hercules, CA, USA) by measuring the average fluorescence intensity, which represented relative expression levels.

### 2.10. Western Blot

Ovarian tissues were homogenized and centrifuged at 12,000× *g* for 10 min to collect the supernatant. Protein concentrations were determined using the BCA assay. Subsequent steps included SDS-PAGE electrophoresis and membrane transfer. Diluted primary antibodies (caspase 3 at 1:1000, cleaved caspase 3 at 1:8000, GSDMD at 1:1000, GSDME at 1:1000, RIPK1 at 1:2000, MLKL at 1:5000, p-MLKL at 1:500, GAPDH at 1:5000) were added and incubated overnight at 4 °C. Following TBST washes, membranes were incubated with secondary antibodies for 1 h at room temperature. Images were captured using a chemiluminescence imaging system, and grayscale analysis was performed using Image-J 1.52i software (Bio-Rad, Hercules, CA, USA).

### 2.11. Statistical Analysis

Data were analyzed using IBM SPSS Statistics 26.0 software (Armonk, New York, NY, USA), and statistical graphs were generated using GraphPad Prism 8.0.2 (San Diego, CA, USA). All data are presented as the mean ± standard error of the mean (SEM). Intergroup comparisons were conducted using one-way analysis of variance (ANOVA) or the Kruskal–Wallis H test, with *p* < 0.05 considered statistically significant. All experiments were conducted with a minimum of three replicates.

## 3. Results

### 3.1. Component Analysis of PMRP

**Figure 1:**
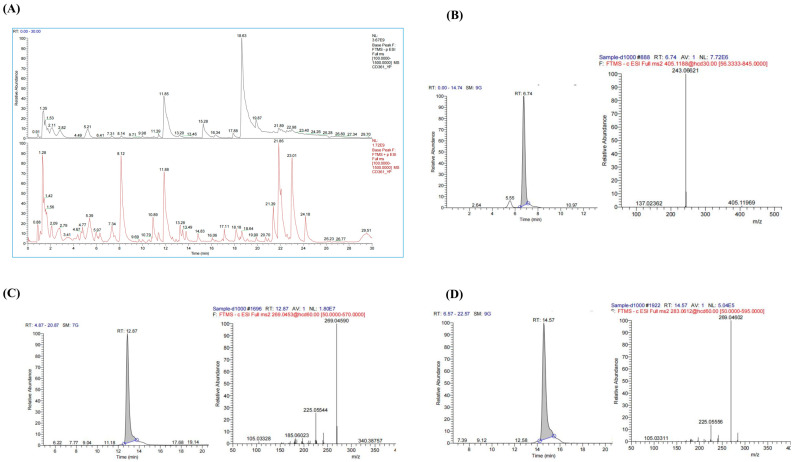
LC–MS analysis of PMRP components. (**A**) Total ion chromatograms showing negative (black) and positive (red) ion modes. (**B**–**D**) Chromatograms and mass spectrums for TSG, Emodin, and Emodin-3-methyl ether.

The chemical composition of PMRP was analyzed using LC-MS, and total ion chromatograms (TIC) were obtained in both positive and negative modes (Figure 1). A total of 38 major compounds were identified, including stilbenes, flavonoids, anthraquinones, and alkaloids (Table S1). For quality control assessment, TSG, Emodin, and Emodin-3-methyl ether were selected for standard comparison and quantification. According to the *Pharmacopoeia of China*, PMRP should contain no less than 0.70% TSG, and the combined content of Emodin and Emodin-3-methyl ether must be at least 0.10% [12]. Our results showed that the TSG content in PMRP was 0.72%, while the total amount of Emodin and Emodin-3-methyl ether was 0.53%, both of which meet the quality control standards for PMRP.

### 3.2. PMRP Improves Estrous Cycle and Ovarian Index in POI Rats

**Figure 2:**
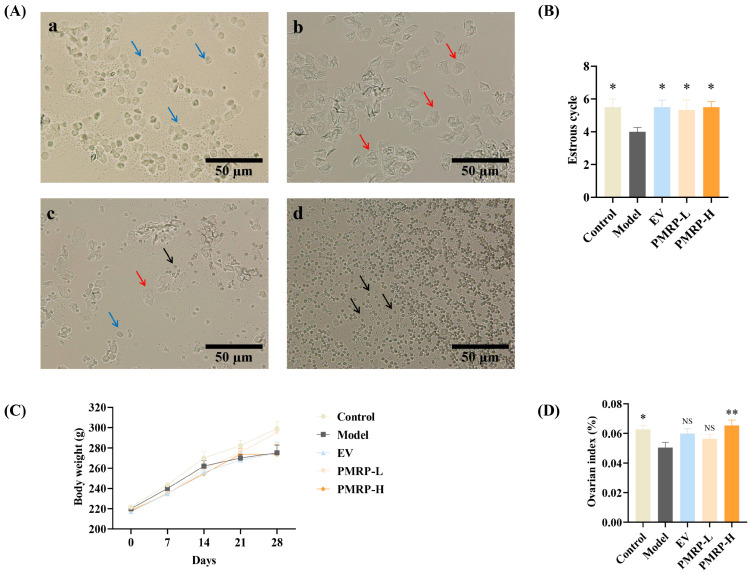
PMRP improves the estrous cycle and ovarian index in POI rats. (**A**) Representative estrous cycle phases (×200): (**a**) Proestrus, (**b**) Estrus, (**c**) Metestrus, (**d**) Diestrus. Blue arrows indicate nucleated epithelial cells, red arrows represent keratinized epithelial cells, and black arrows denote leukocytes. (**B**) Estrous cycle counts. (**C**) Body weight. (**D**) Ovarian index comparison. (Mean ± SEM, n = 6, * *p* < 0.05 and ** *p* < 0.01 vs. model group, NS means not significant).

The normal estrous cycle in rats, spanning 4–5 days, serves as a key indicator of ovarian function (Figure 2A). Vaginal cytology reveals distinct cellular patterns across the cycle phases, as follows: proestrus shows predominantly nucleated epithelial cells, estrus exhibits keratinized epithelial cells, metestrus displays a mixture of epithelial cells and leukocytes, and diestrus is characterized by abundant leukocytes [21]. In this study, while control rats completed approximately 5.5 cycles during the 28-day observation period, the estrous cycles in the model group were markedly reduced (*p* < 0.05) (Figure 2B). Therapeutic intervention with either EV or PMRP (both low and high doses) significantly restored cycle counts (*p* < 0.05). Although all groups showed progressive weight gain, no intergroup differences reached statistical significance (Figure 2C). Moreover, the ovarian index in the model group was significantly lower than that in the control group (*p* < 0.05) and the PMRP-H group (*p* < 0.01) (Figure 2D).

### 3.3. PMRP Regulates Serum Sex Hormone Levels in POI Rats

**Figure 3:**
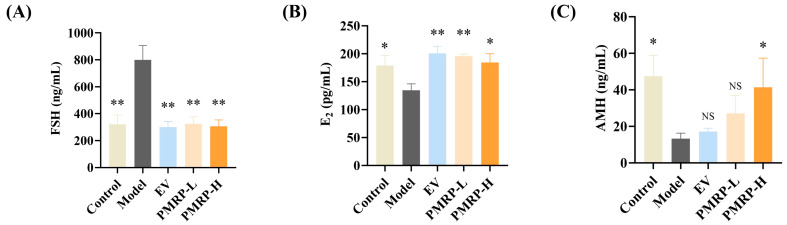
PMRP regulates the serum sex hormone levels in POI rats. (**A**–**C**) Serum concentrations of FSH, E_2_, and AMH. (Mean ± SEM, n = 6, * *p* < 0.05 and ** *p* < 0.01 vs. model group, NS means not significant).

POI is characterized by elevated FSH levels, and decreased E_2_ and AMH levels [2]. Hormonal results indicated pronounced endocrine disruption in POI rats, with the model group exhibiting elevated FSH (*p* < 0.01), alongside reduced E_2_ and AMH levels (*p* < 0.05) relative to the controls (Figure 3A–C). All treatments (EV, PMRP-L, and PMRP-H) significantly ameliorated these hormonal imbalances to varying degrees (*p* < 0.05 or *p* < 0.01).

### 3.4. PMRP Improves Follicular Development in POI Rats

**Figure 4:**
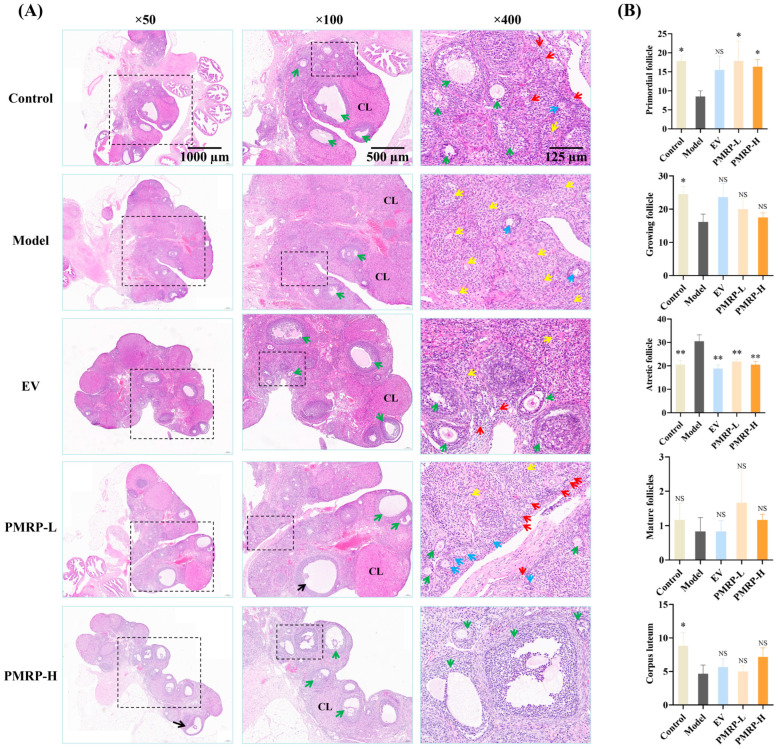
PMRP improves follicular development in POI rats (HE staining). (**A**) Histomorphological alterations in follicles. Red arrows indicate primordial follicles, blue arrows represent primary follicles, green arrows denote secondary follicles, yellow arrows point to atretic follicles, black arrows indicate mature follicles, and CL denotes corpus luteum. (**B**) Numbers of primordial follicles, growing follicles (including primary and secondary follicles), atretic follicles, mature follicles, and corpora lutea. (Mean ± SEM, n = 6, * *p* < 0.05 and ** *p* < 0.01, NS means not significant vs. model group).

The quantity of primordial follicles in the ovarian cortex determines the ovarian reserve. A marked reduction in primordial follicles or a significant increase in follicular atresia can lead to diminished ovarian function [23]. HE staining (Figure 4) demonstrated that the ovarian tissue architecture in the control group was well-preserved, with follicles and corpora lutea distributed appropriately within the cortex. The granulosa cells were arranged in dense multilayered structures, exhibiting abundant primordial follicles and minimal atretic follicles. In contrast, the model group demonstrated a severe loss of granulosa cells, alongside a significant decline in primordial follicles (*p* < 0.05), growing follicles (*p* < 0.05), and corpora lutea (*p* < 0.05), while the number of atretic follicles significantly increased (*p* < 0.01). Therapeutic intervention (EV, PMRP-L, and PMRP-H) resulted in an increased trend of primordial and growing follicles, and significantly reduced atretic follicles (*p* < 0.01). Notably, PMRP-L and PMRP-H groups exhibited superior efficacy in restoring ovarian reserve function, as evidenced by significantly higher numbers of primordial follicles (*p* < 0.05).

### 3.5. PMRP Mitigates PANoptosis-like Death of Granulosa Cells in POI Rats

**Figure 5:**
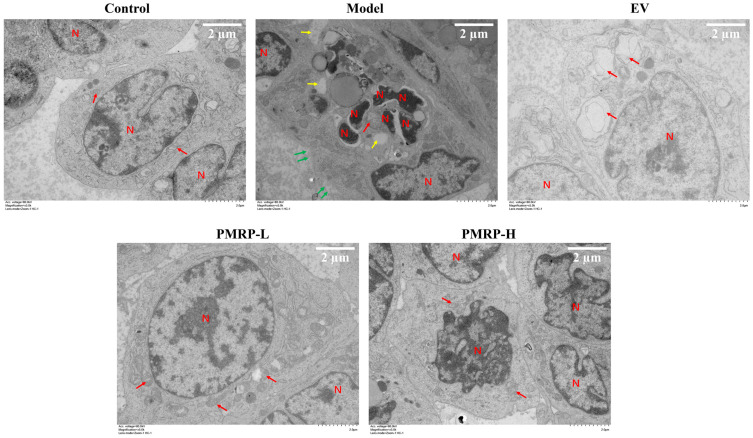
PMRP mitigates the PANoptosis-like death of granulosa cells in POI rats (TEM). Nuclear (N), mitochondria (red arrows), cytoplasmic vacuolization (yellow arrows), and cell membrane rupture with content release (green arrows) are indicated.

TEM analysis (Figure 5) showed that granulosa cells in the control group exhibited normal morphological characteristics, including intact cell membranes, clear mitochondrial cristae, and normally distributed nuclear chromatin. Conversely, granulosa cells from the model group displayed characteristics indicative of apoptosis, pyroptosis, and necroptosis, including chromatin condensation, nuclear fragmentation, cytoplasmic vacuolization, mitochondrial swelling, cell membrane rupture, and cytoplasmic content efflux. These findings suggest that PANoptosis-like death occurred in the granulosa cells of the model group. In the EV group, the distribution of chromatin appeared normal, and the cell membrane remained intact, while the mitochondria displayed swelling with disrupted cristae. The PMRP-L group exhibited nearly normal cellular ultrastructure, with typical mitochondrial architecture and clearly visible cristae. In the PMRP-H group, cells showed partial chromatin condensation but maintained nuclear integrity and predominantly normal mitochondrial morphology. These observations indicate that treatments with EV, PMRP-L, and PMRP-H ameliorate the ultrastructure of granulosa cells in POI rats and mitigate PANoptosis-like cell death.

### 3.6. PMRP Inhibits PANoptosome Assembly in POI Rat Follicles

**Figure 6:**
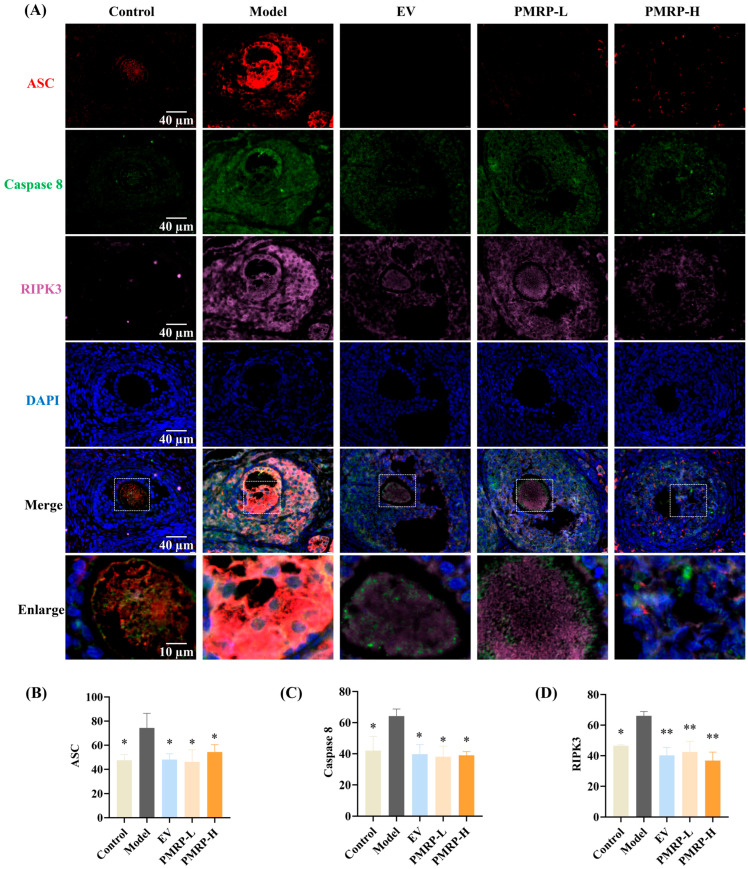
PMRP inhibits the PANoptosome assembly in POI rat follicles. (**A**) Immunofluorescence images of ASC (red), caspase 8 (green), and RIPK3 (rose red) co-localization with nuclear DAPI staining (blue). (**B**–**D**) Average fluorescence intensity of ASC, caspase 8, and RIPK3. (Mean ± SEM, n = 3, * *p* < 0.05 and ** *p* < 0.01 vs. model group). Dashed box shows the zoomed area.

PANoptosis is driven by the PANoptosome, a complex primarily composed of ASC, caspase 8, and RIPK3 [24]. In this study, immunofluorescence was employed to assess the co-localization and relative quantitative expression of ASC, caspase 8, and RIPK3. As depicted in Figure 6, granulosa cells from control rats exhibited minimal expression of these markers and no detectable co-localization. In contrast, the model group displayed pronounced co-localization of ASC, caspase 8, and RIPK3 within primary follicles, secondary follicles, and atretic follicles; the average fluorescence intensity for these markers was significantly higher in the model group compared to the controls (*p* < 0.05), confirming PANoptosome assembly in the growing follicles of POI rats, which contributed to excessive follicle atresia. Therapeutic intervention substantially attenuated this pathological process. All treatment groups (EV, PMRP-L, and PMRP-H) showed markedly reduced co-localization of PANoptosome components compared to the model group. Quantitative assessment revealed significantly decreased fluorescence intensity for all three markers in the EV, PMRP-L, and PMRP-H groups (*p* < 0.05, *p* < 0.01). These results suggest that EV, PMRP-L, and PMRP-H effectively inhibit the PANoptosome assembly in POI rat follicles, thereby preventing PANoptosis.

### 3.7. PMRP Suppresses PANoptosis Effector Proteins in POI Rat Ovaries

**Figure 7:**
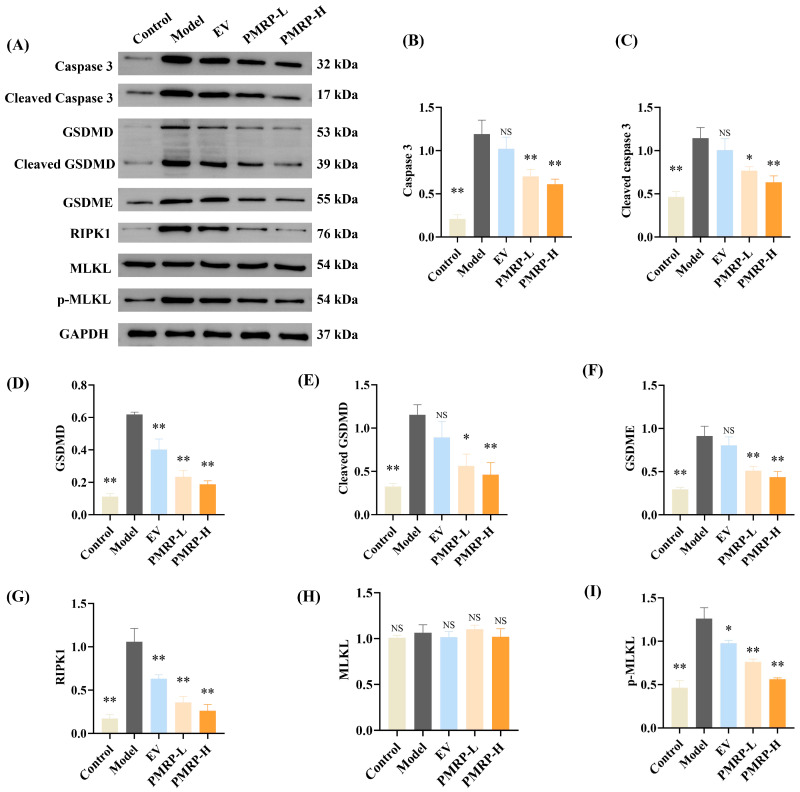
PMRP suppresses PANoptosis effector proteins in POI rat ovaries. (**A**) Western blot images. (**B**–**I**) Relative protein expression levels for caspase 3, cleaved caspase 3, GSDMD, cleaved GSDMD, GSDME, RIPK1, MLKL, and p-MLKL. (Mean ± SEM, n = 3, * *p* < 0.05 and ** *p* < 0.01, NS means not significant vs. model group).

Western blot analysis (Figure 7) was used to evaluate the key effector proteins implicated in PANoptosis. Compared to the control group, ovarian tissues from model group rats demonstrated marked upregulation of multiple effectors, including caspase 3, cleaved caspase 3, GSDMD, cleaved GSDMD, GSDME, RIPK1, and p-MLKL (*p* < 0.01). This comprehensive activation of apoptotic, pyroptotic, and necroptotic pathways confirms the occurrence of PANoptosis in POI rat ovaries. Drug treatments with PMRP significantly decreased the above effector proteins compared to the model group (*p* < 0.05, *p* < 0.01), while EV treatment also effectively downregulated GSDMD, RIPK1, and p-MLKL expression (*p* < 0.05, *p* < 0.01). These results demonstrate that PMRP more comprehensively suppresses PANoptosis effector proteins compared to EV treatment.

## 4. Discussion

PANoptosis, a newly recognized integrated cell death pathway combining features of pyroptosis, apoptosis, and necroptosis, has recently been implicated in various pathological conditions, including infections, sterile inflammation, cancer, and cardiovascular diseases [25,26]. POI represents a significant clinical challenge, associated with both immediate reproductive consequences (e.g., amenorrhea, infertility) and long-term health risks (e.g., osteoporosis, cardiovascular disease) that profoundly affect quality of life. While current management relies heavily on hormone replacement therapy and assisted reproductive technology, these approaches fail to address the underlying ovarian reserve depletion and carry potential long-term risks [10,27]. Traditional Chinese medicine emerges as a promising alternative, given its multi-target approach, fewer side effects, and lower costs. PMRP, a Chinese herb medicine, has been documented for its anti-aging, anti-inflammatory, and immunomodulatory properties [13,14,15]. Previous studies have indicated that PMRP could regulate serum hormone levels and improve ovarian function in rats [16]; however, it remains unclear whether PMRP ameliorates ovarian function by modulating PANoptosis. This study demonstrated a close association between the occurrence of POI and PANoptosis in granulosa cells. Moreover, PMRP treatment significantly inhibited PANoptosis, thereby enhancing ovarian function.

The estrous cycle in normal rats typically spans 4–5 days, with cycle prolongation serving as a reliable indicator of ovarian dysfunction [28]. As established in the research, the biochemical hallmarks of POI consist of elevated FSH, coupled with diminished E2 and AMH levels [2]. Our findings demonstrated that tripterygium glycoside-induced POI resulted in marked estrous cycle elongation and reduced cycle frequency, concurrent with increased serum FSH and decreased E_2_ and AMH concentrations, thereby validating the successful establishment of the model. Therapeutic administration of EV, PMRP-L, and PMRP-H effectively normalized both the estrous cycle and serum sex hormone to levels approximating those observed in healthy controls, confirming their efficacy in restoring the ovarian function in the POI model. Follicles are the fundamental units of the ovary. The orderly activation, development, and natural atresia or maturation of primordial follicles are vital for maintaining the reproductive lifespan. If abundant primordial follicles are activated inappropriately or succumb to death, subsequent follicular atresia accelerates, ultimately leading to ovarian reserve exhaustion and POI onset [23]. Histopathological assessment of POI rat ovaries revealed substantial granulosa cell loss, accompanied by significant reductions in primordial follicles, growing follicles, and corpora lutea, along with increased atretic follicle counts, which further corroborated successful POI induction. Drug treatments notably reduced atretic follicle numbers, indicating therapeutic effects through follicular atresia inhibition. Importantly, PMRP demonstrated superior efficacy in ovarian reserve preservation, as evidenced by increased primordial follicle counts compared to the model group.

Granulosa cells, the primary cell population in follicles, play key roles in follicular development by maintaining an optimal microenvironment for oocyte growth. As the principal producers of ovarian steroid hormones, they are essential for follicular homeostasis. Excessive granulosa cell death disrupts the follicular microenvironment through nutritional deficiencies and metabolic dysregulation, subsequently precipitating oocyte and luteal cell death. This cascade exacerbates follicular atresia, leading to premature ovarian reserve depletion and subsequent POI development [29,30]. TEM revealed that granulosa cells in POI rats displayed concurrent morphological features of apoptosis, pyroptosis, and necrosis, indicative of a PANoptosis-like cell death. These findings suggest that treatments with EV, PMRP-L, and PMRP-H substantially ameliorated the granulosa cell ultrastructure and mitigated PANoptosis-like cell death. Existing evidence suggests PANoptosis may be a key mechanism in ovarian dysfunction [31]. Exposure to short-chain chlorinated paraffins induced ovarian PANoptosis through AIM2- and NLRP12-PANoptosome activation, leading to follicle depletion and hormonal imbalance [9]. Similarly, cranberry-derived exosomes effectively attenuate POF by inhibiting granulosa cell PANoptosis, demonstrating the therapeutic potential of targeting this pathway [32].

PANoptosis represents an inflammatory programmed cell death pathway characterized by PANoptosome complex assembly. This molecular scaffold integrates critical components from multiple cell death pathways, including ASC, caspase-8, and RIPK3 [33,34]. Following activation by specific triggers (e.g., pathogens or stressors), the PANoptosome assembles and activates downstream effectors such as caspase-3, GSDMD/E, and MLKL, ultimately executing cell death [7,35]. Immunofluorescence analysis demonstrated marked co-localization of ASC, caspase-8, and RIPK3 in granulosa cells from both growing and atretic follicles in POI rats, confirming PANoptosome assembly and subsequent PANoptosis activation. This process contributed substantially to excessive follicular atresia. Notably, treatment with EV, PMRP-L, or PMRP-H effectively abolished this co-localization pattern, indicating suppression of PANoptosome assembly and consequent PANoptosis inhibition. Western blot analysis further substantiated these findings, which showed elevated expression of PANoptosis effector proteins in POI rats, including apoptotic molecule caspase-3, cleaved caspase-3, pyroptotic molecule GSDMD, cleaved GSDMD, GSDME, necroptotic molecules RIPK1, and p-MLKL. After the drug intervention, PMRP-L and PMRP-H significantly downregulated the expression of all of these markers, while EV specifically reduced GSDMD, RIPK1, and p-MLKL levels. These results demonstrate the differential capacity of PMRP and EV to inhibit PANoptosis in the POI ovarian tissue, with PMRP showing more comprehensive inhibitory effects across multiple cell death pathways.

While this study provides evidence that PMRP inhibits PANoptosis through protein-level analyses, we acknowledge that RNA-seq profiling would further elucidate the transcriptional networks governing this process. Future studies should characterize the dynamic gene expression changes in granulosa cells following PMRP treatment, particularly regarding upstream regulators of ASC/caspase-8/RIPK3 assembly and downstream effector activation. Such work could identify novel therapeutic targets for POI intervention.

## 5. Conclusions

**Figure 8:**
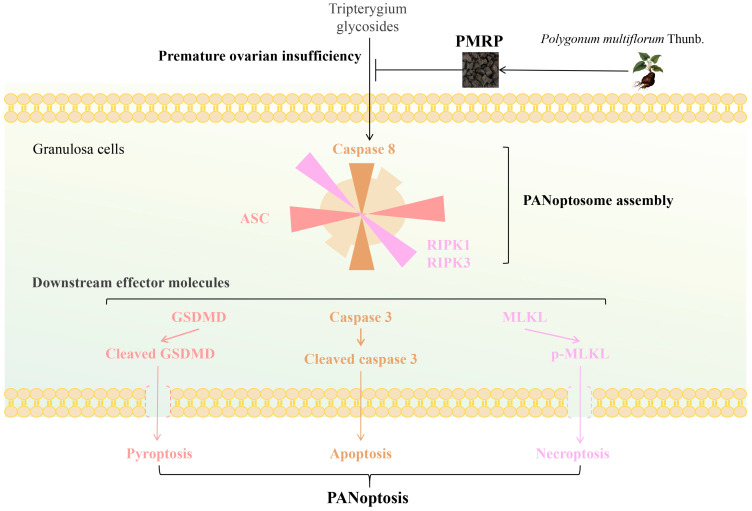
PMRP alleviates POI by inhibiting PANoptosis in follicular granulosa cells.

In conclusion (Figure 8), this study suggests that PMRP effectively suppresses PANoptosis in follicular granulosa cells, thereby reducing follicular atresia and alleviating POI pathology. Our findings overcome the previous limitations associated with targeting isolated death pathways, offering new perspectives for the further exploration of the pathogenesis and treatment strategies for POI.

## Data Availability

The original contributions presented in this study are included in the article/Supplementary Materials. Further inquiries can be directed to the corresponding author/s.

## Supplementary Materials

The following supporting information can be downloaded at: https://www.mdpi.com/article/10.3390/cimb47070569/s1.

## Abbreviations

The following abbreviations are used in this manuscript:

AMH	Anti-Müllerian hormone
AIM2	Absent in melanoma 2
ASC	Apoptosis-associated speck-like protein containing a CARD
E_2_	Estrogen
FSH	Follicle-stimulating hormone
GSDMD/E	gasdermin D/E
HE	Hematoxylin–eosin
LC-MS/MS	Liquid chromatography-tandem mass spectrometry
MLKL	Mixed lineage kinase domain-like protein
NLRP12	NOD-like receptors family pyrin domain containing 12
POI	Premature ovarian insufficiency
POF	Premature ovarian failure
PMRP	Polygoni Multiflori Radix Praeparata
RIPK1/3	Receptor-interacting protein kinase 1/3
SEM	Standard error of the mean
TSG	2,3,5,4′-tetrahydroxystilbene-2-O-β-D-glucoside
TG	Tripterygium glycosides
TEM	Transmission electron microscopy
